# Safety and efficacy of a new spot-on formulation of selamectin plus sarolaner in the treatment and control of naturally occurring flea infestations in cats presented as veterinary patients in Australia

**DOI:** 10.1186/s13071-020-04099-x

**Published:** 2020-05-06

**Authors:** Raj Packianathan, Melissa Pittorino, Andrew Hodge, Natalie Bruellke, Kelly Graham

**Affiliations:** 1 Veterinary Medicine Research and Development, Zoetis Australia Research and Manufacturing Pty Ltd, Level 6, 5 Rider Boulevard, Rhodes, NSW 2138 Australia; 2 Eurofins Animal Health, Unit, F10, 16 Mars Road, Lane Cove West, NSW 2066 Australia; 3 Zoetis, Level 6, 5 Rider Boulevard, Rhodes, NSW 2138 Australia

**Keywords:** Cats, *Ctenocephalides felis*, Ectoparasites, Efficacy, Field study, Flea, Isoxazoline, Parasiticide, Sarolaner, Selamectin, Revolution® Plus, Topical

## Abstract

**Background:**

The safety and efficacy of a new spot-on formulation of selamectin plus sarolaner were evaluated for the treatment and control of natural flea infestations on cats in two non-randomised, multi-centre clinical trials conducted in 8 different locations in Queensland, Australia.

**Methods:**

One hundred and four cats from 65 different households were enrolled across the two studies. Demographic characteristics of cats in the two studies were similar. The new spot-on formulation of selamectin and sarolaner was administered topically once a month for 3 consecutive months at a minimum dosage of 6 mg/kg selamectin (dose range 6–12 mg/kg) plus 1 mg/kg sarolaner (dose range 1–2 mg/kg). Cats were dosed on Days 0 (pre-treatment), 30 and 60 and physical examinations and flea counts were conducted on Days 0, 30, 60 and 90. Efficacy assessments were based on the percentage reduction in live flea counts post-treatment compared to Day 0.

**Results:**

In Study A, at enrolment, primary cats had flea counts ranging from 6 to 107 (arithmetic mean 21.0). The selamectin and sarolaner spot-on formulation resulted in arithmetic mean efficacy of 98.0%, 100% and 100% on Days 30, 60 and 90, respectively. In Study B, at enrolment, primary cats had flea counts ranging from 6 to 22 (arithmetic mean 10.0). The selamectin and sarolaner spot-on formulation resulted in arithmetic mean efficacy of 99.7%, 100% and 100% on Days 30, 60 and 90, respectively.

**Conclusions:**

The new spot-on formulation of selamectin plus sarolaner topically administered at monthly intervals at the minimum dosage of 6.0 mg/kg selamectin and 1.0 mg/kg sarolaner was safe and highly effective against natural infestations of fleas under a range of geographical conditions, representative of both tropical and subtropical regions of Australia.
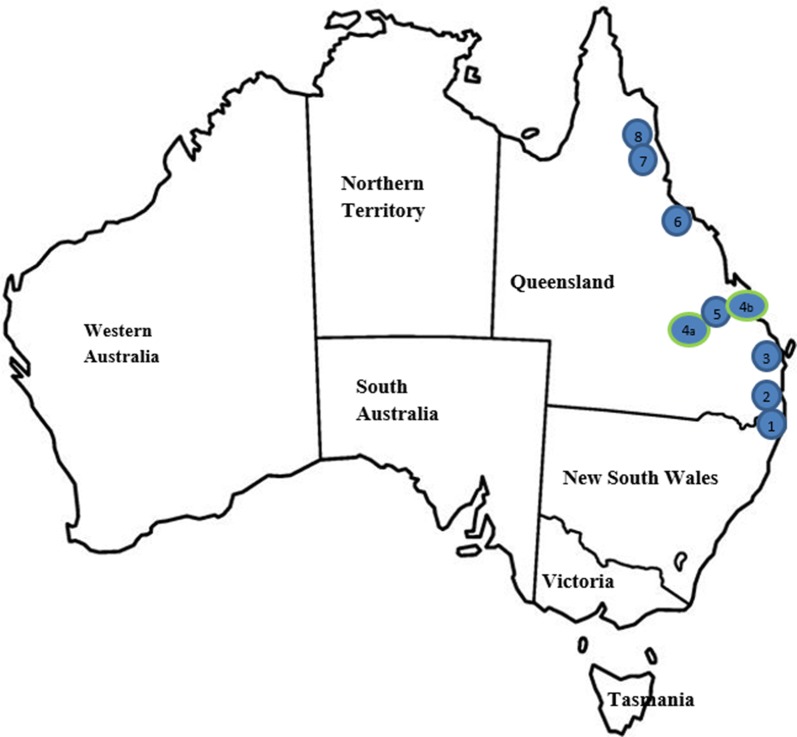

## Background

*Ctenocephalides felis* is the dominant flea species affecting cats in Australia [1–3]. Recent studies have confirmed the genetic diversity among the Australian isolates of *C. felis* with at least three distinct phylogenetic clades identified using mitochondrial DNA markers [4]. Flea infestations cause flea allergy dermatitis, pruritus and anaemia, especially in young cats, but can also transmit a number of zoonotic pathogens such as *Rickettsia felis*, *Bartonella clarridgeiae* [5–8] and *Bartonella henselae* [9]. Flea infestations can occur all year round with a higher incidence during the warmer months. Therefore, flea control throughout the year, including the winter months, is essential to control flea infestation and prevent transmission of pathogens such as *R. felis* [3, 8]. Optimal flea control requires an integrated approach focusing on controlling fleas on the cats as well as controlling flea reproduction in the environment, killing the fleas before they can lay eggs [1, 3, 10, 11]. Historically, various classes of insecticides with different modes of action have been used for flea control. Some of these products also have an extended spectrum of activity against other ecto- and endoparasites in cats [3] including selamectin, as example of a compound in the macrocyclic lactone class of endectocides, which provides effective control against *C. felis* [12, 13], biting lice (*Felicola subrostratus*), ear mites (*Otodectes cynotis*), heartworm (*Dirofilaria immitis*) and intestinal hookworm (*Ancylostoma tubaeforme*) and roundworm (*Toxocara cati*) in cats, but lacks substantial activity against ticks at the labelled dosage [14, 15] .

Sarolaner is an isoxazoline with a wide spectrum of activity against ectoparasites such as fleas, ticks including the Australian paralysis tick, *Ixodes holocyclus* [16], and mites including *Sarcoptes scabiei* var. *canis*, *Demodex canis* and *Otodectes cynotis* in dogs [17, 18]. To broaden the spectrum of activity and provide effective control against both endo- and ectoparasites in cats, a new spot-on formulation was developed with selamectin and sarolaner. This is currently marketed in the USA and Europe as Revolution® Plus or Stronghold® Plus (Zoetis, New Jersey, USA), respectively. The new spot-on formulation has demonstrated efficacy against heartworm [19], fleas [20, 21], various tick species in Europe [22–24] and the USA [25, 26], intestinal hookworms and roundworms [27, 28], and ear mites [29].

Two multi-centric clinical field studies were conducted to evaluate the safety and efficacy of the new spot-on formulation of selamectin plus sarolaner against natural flea infestations in cats presented as veterinary patients under Australian field conditions.

## Methods

The studies were conducted in accordance with the World Association for the Advancement of Veterinary Parasitology (WAAVP) guidelines for evaluating the efficacy of parasiticides for the treatment, prevention and control of flea and tick infestation on dogs and cats [30] and principles of Good Clinical Practice (GCP) [31]. The studies were approved by the Community Access Animal Ethics Committee of the Department of Agriculture and Fisheries Queensland.

### Study locations

**Fig. 1:**
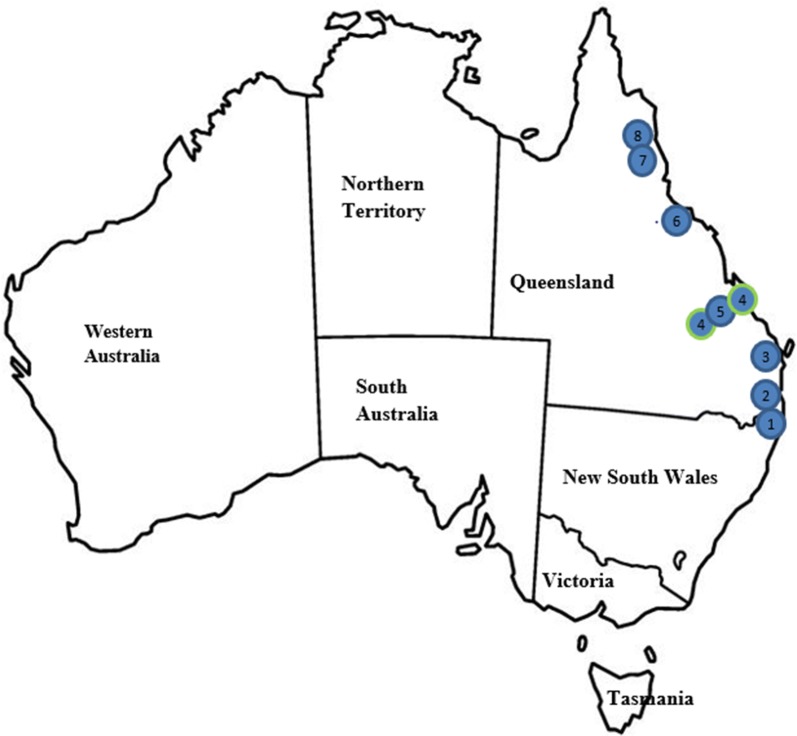
Locations of cats enrolled in two clinical field studies in Australia. Study A Locations (Southern Queensland): 1, Tugun; 2, Paradise Point; 3, Pialba. Study B Locations (Northern Queensland): 4a, Gracemere; 4b, Yeppoon (branch clinics and enrolled as one clinic in the study); 5, Rockhampton; 6, Townsville; 7, Stratford; 8, Mossman

The field studies were conducted in subtropical (Study A: Southern part of Queensland) and tropical (Study B: Northern part of Queensland) regions in Queensland, Australia [32]. The location and number of veterinary clinics where cases were enrolled in the two studies are summarised in Fig. 1.

### Animals

Client-owned cats presented at veterinary clinics or in-house visits by the veterinary investigators were used in the studies. Cats were from diverse households and lived both indoors and outdoors. Cats came from both single cat households and households with multiple cats (maximum of 3 cats) and/or dogs. There were no breed or sex restrictions, but cats intended for breeding or that were pregnant or lactating were not eligible for enrolment. For inclusion in the study, at least one cat in the household had to harbor at least 6 live fleas at screening. All cats were at least 8 weeks of age and ≥ 1.25 kg in body weight at enrolment. Cats in the study were not allowed to have been treated with any ectoparasiticide with persistent activity within 30 days or with a short-acting ectoparasiticide within 14 days of the first treatment. When these studies were conducted, there were no long acting ectoparasiticides in the Australian market for use in cats.

### Experimental design

Both studies followed the same experimental design; however, studies A and B were analysed and reported separately, as they represented different geographical regions. Each study was conducted as a multi-centric, single group, non-randomised trial. Within each household, the primary cat was the first cat in the household with ≥ 6 fleas counted on Day 0 and up to 2 additional cats were enrolled as supplementary cats. Only the primary cats were included in the efficacy evaluation whereas all cats were included in the safety evaluation.

All cats were confirmed to be in good general health prior to enrolment based on the physical examination performed by a veterinarian. Cats were housed and maintained under their normal home conditions for the duration of the study. No additional products that had activity against fleas (including systemic, premises, and/or over-the-counter treatments including insecticidal shampoos or collars) were permitted to be used on any animal in the household for the duration of the study. Any concomitant medications used during the study were recorded along with any abnormal health events.

### Treatment administration

Cats received three consecutive monthly treatments on study days 0, 30 and 60. Day 0 was set as the day the primary cat in each household received the first treatment. For the follow-up treatments on Days 30 and 60, the visits were allowed to deviate by ± 3 days of the target date. Treatments were dispensed by the veterinarian. Treatment dispensing was based upon the most recent body weight recorded prior to each treatment. Cats were topically dosed with the recommended label dosage of the spot-on formulation containing 6.0–12.0 mg/kg selamectin and 1.0–2.0 mg/kg sarolaner. After each treatment, cats were not permitted to be bathed for 24 hours or groomed throughout the study.

### Flea counts

Flea counts on primary cats were conducted prior to treatment on Days 0, 30 and 60 as well as on Day 90 (the post-treatment evaluations could be conducted ± 3 days of the target day) by a qualified and/or experienced veterinary staff.

Flea counts were conducted by personnel trained to a standardized methodology. The cats were combed with a fine-toothed flea comb that was uniquely identified for each cat. The combing proceeded in a systematic manner to ensure all areas of the cat were combed. Each cat was examined for at least 10 min. If any fleas were found, the examination was continued in 1-min increments until no fleas were encountered. Once combing was completed, all fleas were placed back on the cat. Fleas maintaining an upright orientation or moving in a coordinated manner were considered to be alive. Only live flea counts were recorded.

### Statistical analysis

Data were summarised and analysed for each of the two studies separately, using SAS version 9.3 (SAS Institute Inc., Cary, NC, USA). The individual animal (primary cat) was the experimental unit for the efficacy analysis. Data were excluded from the efficacy analysis following protocol deviations such as incorrect dosing or where dosing or flea counts were not conducted within ± 3 days of the target day (after Day 0). Flea counts were summarized at each time point using arithmetic means and ranges. Percent effectiveness with respect to the baseline was calculated at each time point using the formula [(C − T)/C] × 100, where C is the pre-treatment arithmetic mean and T is the post-treatment arithmetic mean.

## Results

### Demographics

Enrolment and demographic characteristics are summarised in Table 1.

**Table 1:** Demographic characteristics of primary cats enrolled in two clinical field studies in Australia and treated with selamectin (6–12 mg/kg) plus sarolaner (1–2 mg/kg)

Characteristic	Study A (3 clinics, *N* = 31)	Study B (5 clinics, *N* = 34)
	*n* (%)	*n* (%)
Breed		
Purebred	1 (3)	2 (6)
Non-purebred	30 (97)	32 (94)
Living condition		
Indoors and outdoors	17 (55)	15 (44)
Mostly indoors	9 (29)	13 (38)
Mostly outdoors	5 (16)	6 (18)
Sex		
Male	17 (55)	19 (56)
Female	14 (45)	15 (44)
Neutered		
Yes	24 (77)	16 (47)
No	7 (23)	18 (53)
Hair type		
Long	5 (16)	2 (6)
Medium	6 (19)	7 (21)
Short	20 (65)	25 (74)

#### Study A

A total of 31 primary cats were enrolled across 3 different clinics in the southern part of Queensland. Most of the primary cats were crossbred (*n* = 30; 97%) and most were neutered (*n* = 24; 77%), with similar numbers of males (*n* = 17) and females (*n* = 14). Out of 31 households, 10 were single cat households and 21 had additional dogs or cats; 11 households had both dogs and cats. All dogs in the households were treated for fleas with ‘Nexgard (afoxolaner) Chewable for dogs’ (Boehringer Ingelheim Pty Ltd, New South Wales, Australia). The ages of the primary cats ranged from 10 weeks to 13 years, with a mean age of 4.7 years and weighed 1.3–7.8 kg. Eighty-four percent (*n* = 26) of primary cats had short or medium hair length and the majority (*n* = 22; 71%) had outdoor access, with only 29% (*n* = 9) living mainly indoors. Of the 31 primary cats, 2 cats did not complete the study due to owner non-compliance.

#### Study B

A total of 34 primary cats were enrolled across 5 different clinics (including mobile veterinary clinic investigator visiting households) in the northern part of Queensland. Of the 34 primary cats, 2 (6%) were purebred and 32 (94%) were crossbred. Approximately half of the primary cats were neutered (*n* = 16; 47%), with similar numbers of males (*n* = 19) and females (*n* = 15). The ages of the primary cats ranged from 10 weeks to 13 years, with a mean age of 3.2 years and weighed 1.3–6.5 kg. Ninety-four percent (*n* = 32) of primary cats had short or medium hair length and the majority (*n* = 21; 62%) had outdoor access, with only 38% (*n* = 13) living mainly indoors. Out of 34 households, 9 were single cat household only and 25 had additional dogs or cats; 23 households had both dogs and cats. All dogs in the households were treated for fleas with ‘Nexgard (afoxolaner) Chewable for dogs’ (Boehringer Ingelheim Pty Ltd). Of the 34 primary cats, six cats did not complete the study due to owner non-compliance and other reasons.

### Safety

In study A and B, a total of 55 and 49 and cats were enrolled for safety evaluation, respectively. A total of nine adverse events in Study A and two in Study B were recorded. Most of the observed adverse events in both studies were sporadic in nature and typical of those commonly seen in the general cat population such as skin infections and gastrointestinal and ocular disorders. One cat (6 year-old, neutered female) in Study A was reported to have vomiting from 2 hours after the second monthly treatment application and vomiting continued for 4 days and the cat was presented at the veterinary clinic for examination 4 days after treatment. Physical examination, blood biochemistry and complete blood counts were performed, and no clinically significant findings were noted. The cat was treated with maropitant injection and the condition resolved without further treatment. Since there was no other attributable cause of vomiting, the vomiting episode was deemed to possibly be related to the treatment. There were no other adverse events assessed as treatment-related in any of the other selamectin and sarolaner spot-on formulation-treated cats.

### Efficacy

#### Study A

At enrolment, primary cats had flea counts ranging from 6 to 107 (arithmetic mean = 21.0). The selamectin and sarolaner spot-on formulation resulted in arithmetic mean efficacy of 98.0%, 100% and 100% on Days 30, 60 and 90, respectively (Table 2).

**Table 2:** Flea counts, ranges of counts and arithmetic mean (AM) efficacy at each time point for primary cats treated with selamectin (6–12 mg/kg) plus sarolaner (1–2 mg/kg) in two clinical field studies in Australia

Day of study	Study A	Study B
Day 0		
No. of animals	31	34
Arithmetic mean count	21.0	10.0
Range of counts	6–107	6–22
Day 30		
No. of animals	29	29
Arithmetic mean count	0.4	0.0
Range of counts	0–4	0–1
AM efficacy (%)^a^	98.0	99.7
Day 60		
No. of animals	27	28
Arithmetic mean count	0.0	0.0
Range of counts	0–0	0–0
AM efficacy (%)^a^	100	100
Day 90		
No. of animals	21	26
Arithmetic mean count	0.0	0-0
Range of counts	0–0	0–0
AM efficacy (%)^a^	100	100

^a^Efficacy calculated based on comparison to arithmetic mean on Day 0

#### Study B

At enrolment, primary cats had flea counts ranging from 6 to 22 (arithmetic mean = 10.0). The selamectin and sarolaner spot-on formulation resulted in arithmetic mean efficacy of 99.7%, 100% and 100% on Days 30, 60 and 90, respectively (Table 2).

## Discussion

One hundred and four cats from 65 different households were enrolled across the two studies in the subtropical and tropical regions of Australia (Fig. 1). Demographic characteristics of cats in the two studies were generally similar. The new spot-on formulation of selamectin and sarolaner administered topically once a month for 3 consecutive months at a minimum dosage of 6 mg/kg (dose range 6–12 mg/kg) selamectin plus 1 mg/kg (dose range 1–2 mg/kg) sarolaner resulted in excellent treatment and control of naturally occurring flea infestations on client-owned cats. The new spot-on formulation of selamectin and sarolaner was well tolerated, with the observed abnormal clinical signs consistent with conditions most commonly seen in the general cat population and not related to study treatment, except in one cat where vomiting was possibly related to the treatment.

The treatment with selamectin and sarolaner spot-on provided 98.0% (Study A) and 99.7% (Study B) mean efficacy at 30 days after the first treatment and these findings were consistent with previous data reported in overseas field studies in the USA and Europe [20, 22] where following monthly administration on Days 30 and 60, the new spot-on formulation provided 100% efficacy against fleas for 30 days as reported previously [20–22]. Under field conditions, cats are continuously exposed to new flea infestations and therefore the rapid speed of kill is critical in reducing the flea burden on the cats as well as breaking the flea life-cycle and halting their development in the environment [3, 33].

Although identification of fleas in the field was not conducted in these studies, it is well documented that *C. felis* is the most common cause of flea infestations in cats in Australia [2]. Using the mtDNA sequencing of cytochrome *c* oxidase subunits, different haplotype clades of *C. felis* were identified in different parts of Australia [34]. Although the insecticidal efficacy against different haplotype clades of *C. felis* is unknown, a proportion of enrolled cats in both studies (Fig. 1) came from the regions where the different haplotype clades of *C. felis* have been reported [34].

*Ctenocephalides felis* has been reported to develop resistance to some of the older classes of ectoparasiticides [1], but the lack of or reduced efficacy of flea products reported in the field is generally most likely due to poor owner compliance, high environmental flea burden, poor understanding of flea biology or different susceptibility of fleas [3, 10, 13, 35]. Inclusion of sarolaner in the new spot-on formulation will complement the pulicidal activity of selamectin as well as provide effective control against ticks [22–24]. The new spot-on formulation was also demonstrated to have rapid speed of kill following administration thus killing the fleas before they can lay eggs [20, 21], and was effective in reducing the environmental flea burden [36] thereby reducing the risk of spreading vector-borne diseases caused by *R. felis* and *Bartonella* spp. [5–9, 37].

## Conclusions

The new spot-on formulation of selamectin plus sarolaner topically administered at monthly intervals at the minimum dosage of 6.0 mg/kg selamectin and 1.0 mg/kg sarolaner, was well tolerated and highly effective against natural infestations of fleas under a range of Australian conditions.

## Data Availability

Relevant datasets generated and/or analysed during these studies are included within the article.
